# A Peculiar Cause of Shock: Analysing Two Clinical Cases

**DOI:** 10.1155/2023/8901383

**Published:** 2023-12-02

**Authors:** João Oliveira, Alberto Costa-Silva, Luís Vale, Daniel Costa, Rui Almeida-Pinto, Carlos Martins-Silva, Tiago Antunes-Lopes, João Silva

**Affiliations:** ^1^Urology Department, Centro Hospitalar Universitário São João, Porto, Portugal; ^2^Faculty of Medicine, University of Porto, Porto, Portugal

## Abstract

**Introduction:**

Pheochromocytoma is a rare neoplasia arising from the adrenal medulla that secretes catecholamines. Those afflicted by this condition can present a wide range of symptoms. One of the most common is paroxysmic hypertension. Interestingly, although rare, some patients present with shock. We describe two cases of pheochromocytoma in which the initial presentation was shock. *Case 1*. 49 year-old woman, with a history of resistant hypertension, presented to the emergency department with thoracic pain and fever. EKG, echocardiogram (ECC), and myocardial necrosis markers were compatible with Takotsubo syndrome (TS). CT demonstrated a staghorn calculus, hydronephrosis, and signs compatible with xanthogranulomatous pyelonephritis in the right kidney. Additionally, and incidentally, it revealed a 60 mm nodule on the right adrenal gland. Piperacillin/tazobactam was started immediately, and the patient was submitted to urgent upper urinary tract drainage. This procedure was complicated by a cardiorespiratory arrest that was treated with adrenaline administration. The patient was admitted to the ICU due to multifactorial shock and started alpha and, posteriorly, beta blockage. Biochemical adrenal incidentaloma endocrinologic study was negative (under hemodialysis). Multiorgan failure progressively improved. After 2 weeks, the patient was submitted to a laparoscopic transperitoneal right adrenalectomy. No complications were reported. Histological analysis revealed a pheochromocytoma. *Case 2*. 28-year-old woman presented to the emergency department with headaches and nausea. Vitals were compatible with shock. CT revealed an incidental 72 mm mass on the right adrenal. EKG, ECC, and myocardial necrosis markers were compatible with TS. The patient was started on alpha and, posteriorly, beta blockage. Adrenal incidentaloma endocrinological study demonstrated high urinary catecholamines. Right transperitoneal adrenalectomy was performed. No complications were noted. Histological analysis revealed a pheochromocytoma.

**Conclusion:**

Pheochromocytoma can present with complex, enigmatic, and rare clinical pictures. Clinicians should be wary of the possibility of this diagnosis when managing adrenal masses.

## 1. Introduction

Pheochromocytoma (PC) is a neuroendocrine neoplasia of the adrenal medulla that produces and secretes catecholamines [1]. It is a rare condition, affecting 0.6 per 100000 persons per year. Nevertheless, incidence has been increasing, due to improved diagnosis through better and more available imaging tools and wider clinical awareness [2]. Classically, patients present with signs and symptoms attributable to catecholamine excess, namely, palpitations, tachycardia, chest pain, hyperhidrosis, and headaches [3].

Shock is a life-threatening condition characterized by poor peripheral perfusion, implicating cellular and tissue hypoxia. Several conditions can precipitate shock [4].

Nonetheless, it is a very uncommon presentation of pheochromocytoma, with only few cases reported in literature [5, 6].

Therefore, we aim to describe two cases of pheochromocytoma presenting with shock at admission to the emergency department [7].

## 2. Case Presentation

### 2.1. Case 1

A 49-year-old woman presented to the emergency department with fever, headache, nausea, and vomiting for 12 hours. She had a history of refractory hypertension and was concomitantly prescribed with lisinopril, hydrochlorothiazide, and lercanidipine. She denied any other relevant comorbidities or family history.

On arrival, the patient's blood pressure was labile (first measurement 99/51 mmHg; maximum registered 181/103 mmHg), she was tachycardic (105 bpm) and febrile (temperature 38.9°C) and presented signs of poor peripheral perfusion.

Arterial blood gas analysis found pH 7.357, pO2 77.9 mmHg, pCO2 31.0 mmHg, and HCO3-19.6 mmol/L and demonstrated hyperglycemia (glucose 448 mg/dL) and lactic acidosis (lactate 6.5 mmol/L - reference range < 1 mmol/L), compatible with impaired peripheral perfusion. Laboratory workup revealed leukocytosis (24^∗^10^9^/L; 90% neutrophils) and thrombocytopenia (60∗10^9^/L). Plasma creatinine was 1.78 mg/dL, C-reactive protein was 66 mg/dL, and troponin was 464 ng/L. Full blood sample analysis can be found in Table 1. We obtained urine and blood cultures and started empiric antibiotic therapy using piperacillin/tazobactam (IV 4.5 mg every 6 h).

**Figure 1:**
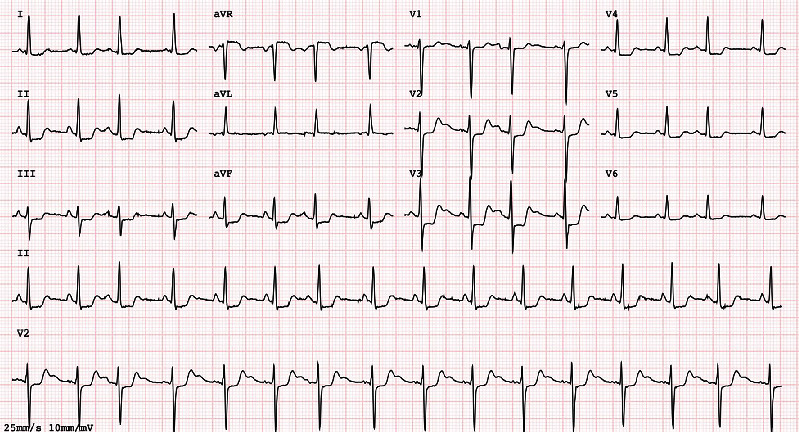
Case 1 EKG (at admission). EKG identified sinus tachycardia and diffuse ST segment depression.

EKG identified sinus tachycardia and diffuse ST segment depression, as can be analyzed in Figure 1. The echocardiogram demonstrated apical hypercontractility with basal akinesia. Taken together with the clinical picture of shock, these findings were suggestive of *Takotsubo* syndrome.

**Figure 2:**
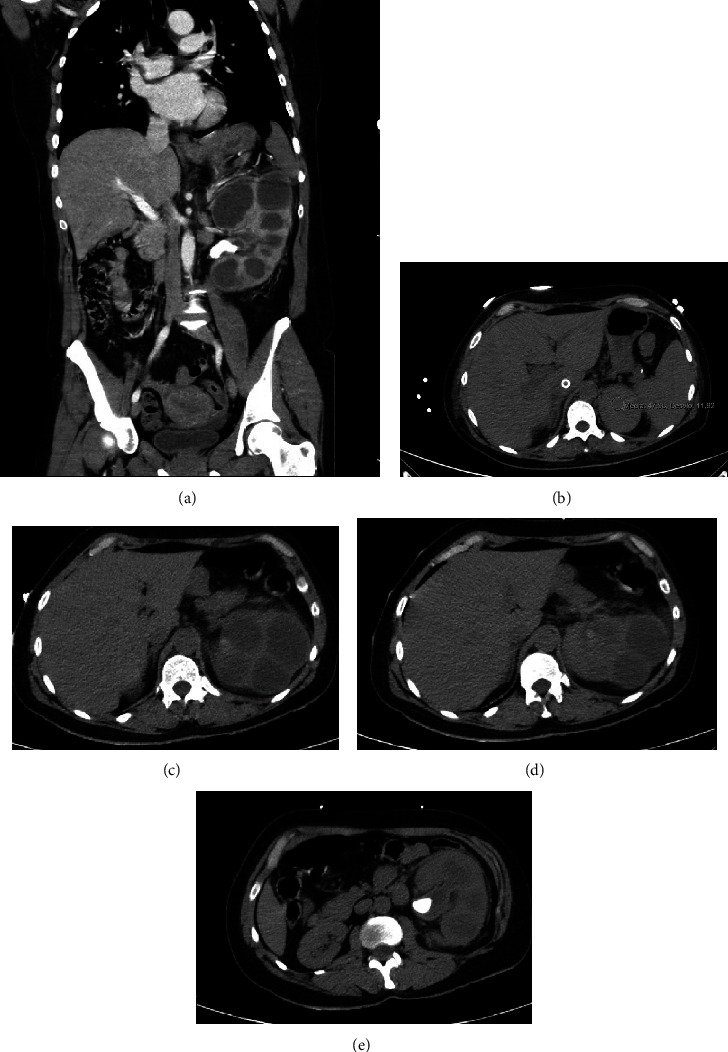
Case 1 thoracoabdominopelvic CT scan (at admission). CT scan revealed an incomplete staghorn calculus measuring 26∗14 mm impacted in the right pyelo-junction inducing hydronephrosis (a, e) and signs compatible with xanthogranulomatous pyelonephritis (a, c–e). A solid and heterogeneous right adrenal mass of 60∗55∗62 mm can also be identified (b).

In the face of the obscure septic focus, we obtained a thoracoabdominopelvic CT scan. Images demonstrated an incomplete staghorn calculus measuring 26∗14 mm impacted in the right pyeloureteral junction inducing hydronephrosis and signs compatible with xanthogranulomatous pyelonephritis. A solid and heterogeneous right adrenal mass of 60∗55∗62 mm was also identified. CT images are depicted in Figure 2.

Patient's clinical status deteriorated swiftly, developing type 2 respiratory failure requiring endotracheal intubation and mechanical ventilation. Emergent upper urinary tract drainage with double J stent placement was performed. During the procedure, the patient suffered a cardiorespiratory arrest and reverted with adrenaline administration and chest compressions.

**Figure 3:**
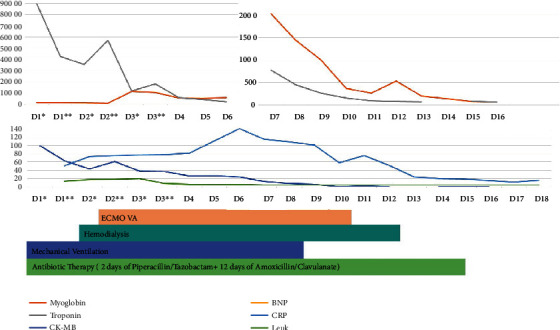
Case 1 clinical and analytical evolution. Case 1 analytical evolution of cardiac necrosis markers and inflammatory markers. The patient was under ECMO between the 2^nd^ and 10^th^ days, hemodialysis between the 2^nd^ and 12^th^ days, and mechanical ventilation until the 8^th^ day. She was treated with 14 days of effective antibiotic therapy 2 days of piperacillin and tazobactam and 12 days of amoxicillin/clavulanate. BNP: B natriuretic peptide; CK-MB: creatine kinase-myocardial band; CRP: C-reactive protein; Leuk: leucocytes. In the first days in the ICU, two blood sample analyses were performed daily, which are depicted in the graph as morning (^∗^) and evening (^∗∗^). For graphical coherence, the troponin and myoglobin level scale is at a tenth of the real value.

She was admitted to the ICU under continuous hemodialysis, vasopressor support, and mechanical ventilation. Nevertheless, persistent hemodynamic instability culminated on the initiation of venous-arterial extracorporeal membrane oxygenation (ECMO) by the second day. Owing to pheochromocytoma suspicion, she was started on alpha-adrenergic blockage on the second day and beta blockage at the sixth day. Urinary catecholamines at the second day were within the reference range (patient was under hemodialysis at that time). Plasma dehydroepiandrosterone sulfate (DHEAS), adrenocorticotropic hormone (ACTH), and urinary 24-hour cortisol were also unremarkable. Patient's clinical status improved progressively. Clinical evolution and analytical results can be found schematically in Figure 3. She was treated with 14 days of effective antimicrobial therapy: 2 days of piperacillin/tazobactam and 12 days of amoxicillin-clavulanate, after urine culture isolated an *E. coli* susceptible to the latter.

**Figure 4:**
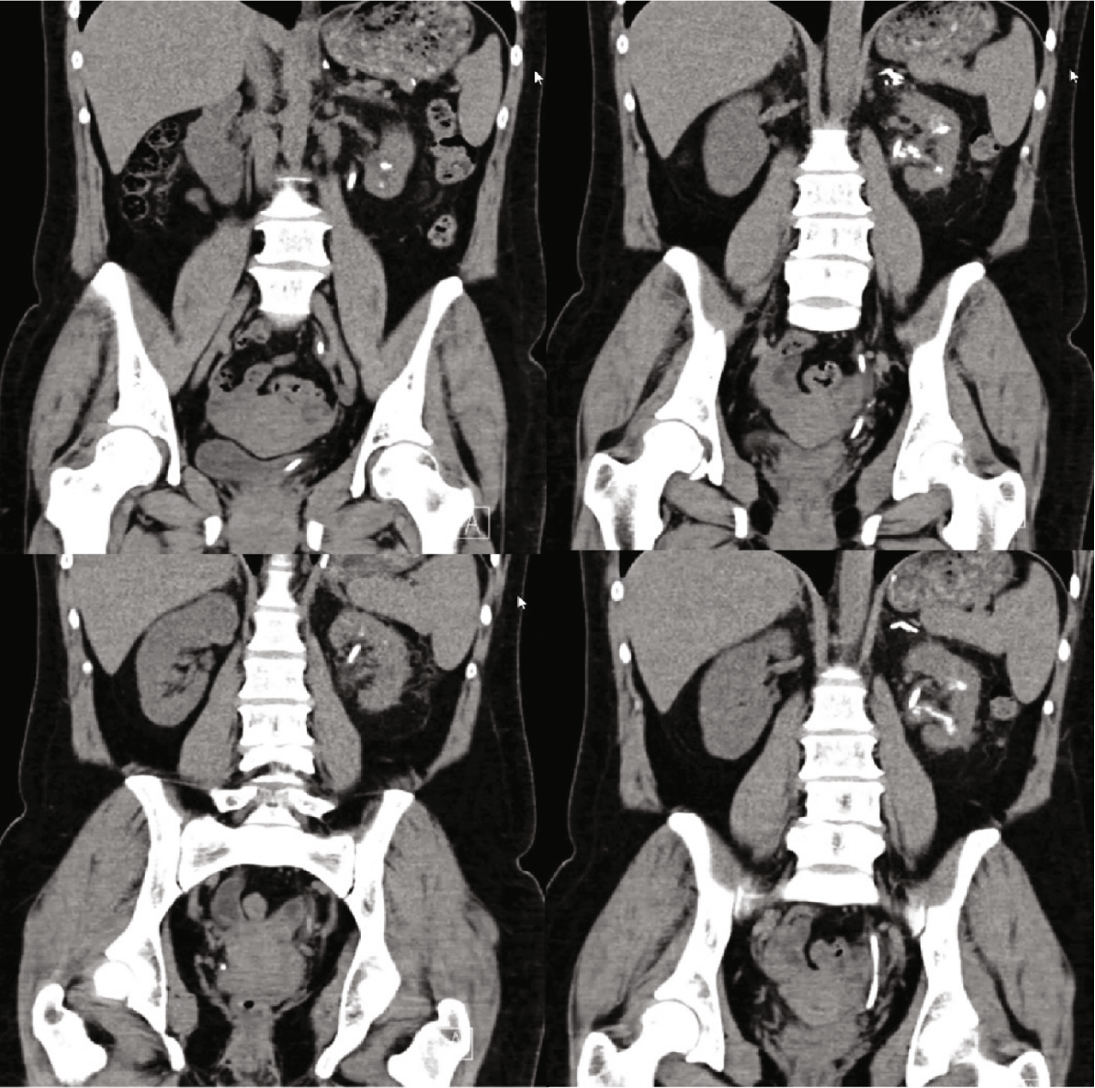
Case 1 abdominopelvic CT scan (after flexible RIRS with thulium laser). The CT scan shows residual lithiasis of small size (including intraparenchymal stones) and a double J stent.

By the 21^st^ day, we performed a laparoscopic transperitoneal right adrenalectomy. No complications were registered. Pathology analysis of the removed specimen identified a pheochromocytoma. The patient was discharged home at the 36^th^ day. After 3 months, the patient was submitted to retrograde intrarenal surgery (RIRS) with thulium laser stone fragmentation in two sessions with an excellent postoperative result, as can be confirmed by the CT images available in Figure 4.

### 2.2. Case 2

A 28-year-old woman with a previous history of nephrolithiasis was admitted to the emergency department with headache, nausea, and vomiting. She denied any other personal or family history On arrival, she was hypoxemic (O2 saturation 66%) and tachycardic (155 bpm). Blood pressure was 98/57 mmHg, and temperature was 35.9°C.

**Figure 5:**
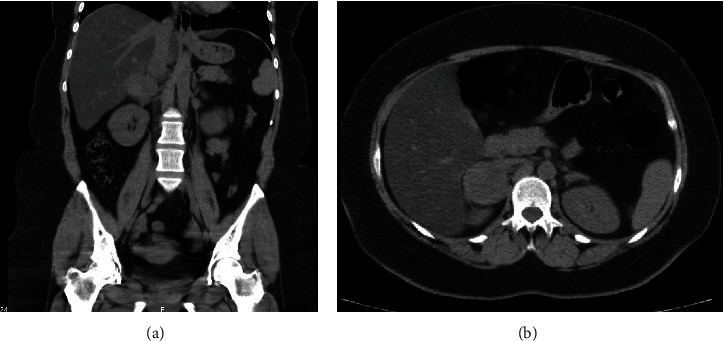
Case 2 abdominopelvic CT scan (at admission). The CT scan shows a 72∗66 mm bilobate mass of the right adrenal gland in coronal (a) and transverse (b) views.

Arterial blood gas analysis showed severe hypoxemia (pO2: 37 mmHg), hypocapnia (pCO2: 28 mmHg), and lactic acidosis (lactate 7 mmol/dL). Laboratory workup identified elevated serum troponin levels (26147 ng/L), but normal renal function (urea 59 mg/dL; creatinine 1 mg/dL). Full blood sample analysis can be found in Table 2. EKG depicted sinus tachycardia and diffuse ST segment depression. Echocardiogram unveiled diffused hypokinesia sparing only the apex, compatible with *Takostubo* syndrome. Thoracoabdominopelvic CT scan revealed a 72∗66 mm bilobate mass of the right adrenal gland. CT images are reproduced in Figure 5.

**Figure 6:**
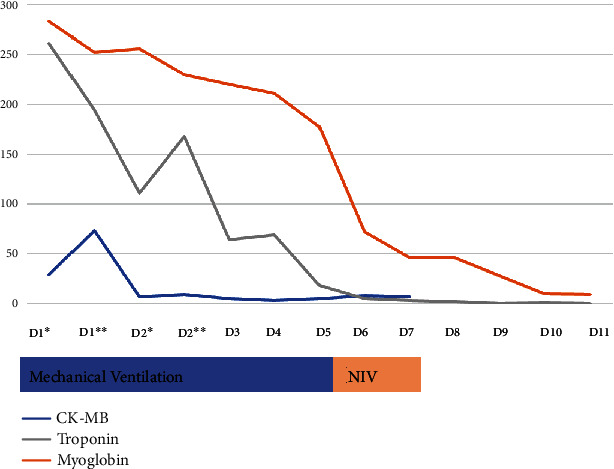
Case 2 clinical and analytical evolution. Case 2 analytical evolution of cardiac necrosis markers. The patient was under mechanical ventilation until the 5^th^ day and between the 5^th^ and 7^th^ day under noninvasive ventilation. CK-MB: creatine kinase-myocardial band; NIV: noninvasive ventilation. In the first days in the ICU, two blood sample analyses were performed daily, which are depicted in the graph as morning (^∗^) and evening (^∗∗^). For graphical coherence, troponin and myoglobin level scale is at a tenth of the real value.

Due to the multiorgan failure in the context of the cardiogenic shock, the patient was admitted to the ICU under mechanical ventilation and aminergic support. Considering a suspected diagnosis of pheochromocytoma, she was started on alpha blockage by the 5^th^ day, and three days later on beta blockage. Urinary catecholamine levels were elevated (norepinephrine 2494.55 ug/24 h; epinephrine 260.52 ug/24 h, dopamine 571.29 ug/24 h). Plasma DHEAS, ACTH, and urinary 24-hour cortisol were within the normal reference range. Patient clinical status improved progressively allowing discharge from the ICU to the urology ward by the 16^th^ day. Clinical evolution and analytical results can be found schematically in Figure 6.

At the 21st day, she was submitted to a laparoscopic transperitoneal right adrenalectomy, with no intra- or postoperative complications. Pathological analysis of the removed specimen confirmed the diagnosis of a pheochromocytoma. She was discharged at the 8^th^ day after surgery.

## 3. Discussion

Pheochromocytoma is a neuroendocrine neoplasia arising from the chromaffin cells of the adrenal medulla that produce and release catecholamines. This is a rare disease, affecting only 0.1-0.6% of patients undergoing screening for secondary hypertension and 0.05% in autopsy reports [2]. Nevertheless, in the group of patients with an adrenal mass, prevalence reaches 5% [8].

The classic symptom triad associated with this condition includes headaches, tachycardia, and chest pain. Notwithstanding, currently, most patients are diagnosed in the context of secondary hypertension investigation. There is also an important subset of asymptomatic patients diagnosed during the investigation of an adrenal incidentaloma or even remaining undiagnosed. This is cleverly illustrated by a study analysing 54 autopsy-proven PCs, reporting that 76% of them were only diagnosed postmortem. Pheochromocytomas can afflict patients of all ages, albeit peak incidence occurs between the 4^th^ and 5^th^ decades [9, 10].

PC diagnosis implies an excess of catecholamines and a compatible image. The workup includes an abdominal CT imaging and endocrinologic examination [11]. CT imaging allows tumor identification and careful surgical planning. Regarding catecholamine level measurement, several methodologies exist, such as 24-hour urinary metanephrine or catecholamine determination, and plasma fractionated metanephrine dosing. Nonetheless, in some patients, catecholamine excess cannot be demonstrated either because they are diagnosed in the prebiochemical stage or due to limitations of the diagnostic methods. Urinary 24-hour metanephrine determination is the most common available test, although it has some limitations as the possibility of false negative results in patients with renal failure on dialysis [11]. This was the case of the first patient.

Adrenalectomy is the treatment of choice for localized pheochromocytoma. This procedure may be performed by either an open or laparoscopic approach. The choice between the latter is based on the size of the mass and the surgeon/center experience. Current guidelines recommend the laparoscopic approach for masses measuring up to 6 cm. Nevertheless, there is evidence suggesting comparable surgical outcomes that can be achieved for bigger masses in centers with elevated laparoscopic experience, as is the case presented [11].

*Takotsubo* syndrome (TTS), also known as stress cardiomyopathy, is a cause of acute reversible cardiac failure, first described in Japan in the 90s. It is a rare disease characterized by apical ballooning of the left ventricle, mimicking the image of a *Takostubo* octopus pot, due to its broad base and narrow neck [12]. TTS affects 1-2% of troponin-positive suspected acute coronary syndromes or suspected ST-segment elevation myocardial infarction and is more common in women than in men. TTS incidence in pheochromocytoma patients is reported up to 3% [13].

Patients typically present with substernal chest pain and, in some cases, with signs of cardiogenic shock. In the case of PC patients, the latter is the most common presentation of this entity. This type of symptomatology evokes an important differential diagnosis with acute coronary syndrome [14].

EKG abnormalities are common in TCC: most frequently ST segment elevation, or ST segment depression, although the latter affects less than 10% of cases. Myocardial necrosis markers are generally elevated: for instance, troponin levels can rise up to 7 times above the upper limit [14].

*Takostubo* syndrome can be subdivided in two echocardiographic phenotypes: typical or atypical. Typical involves apical ballooning of the left ventricle and basal hyperkinesis. Atypical includes several abnormalities such as apical hyperkinesis and mid ventricular ballooning. Atypical syndrome has been reported to be more frequent in younger patients. In this paper, we report a case of a typical TSS (case 1) and another of an atypical TSS (case 2) [15].

Several diagnostic criteria for TSS have been proposed over the years. InterTAK diagnostic criteria developed by the Takotsubo International Registry are the most recently published [16]. These criteria differ from the previous as they recognize that coronary heart disease and TSS can coexist and acknowledge the role of pheochromocytoma as a TSS trigger, as described in this paper. Therefore, according to the latter, a pheochromocytoma diagnosis is no longer against a TSS diagnosis.

Pathophysiology involves catecholamine excess that can be caused iatrogenically by catecholamine administration. Furthermore, adrenaline levels are higher in TSS patients when compared with those suffering from acute coronary syndrome. This might explain TSS occurrence in a subset of pheochromocytoma patients [17]. Furthermore, there is an intricate relationship between the steroid hormones and catecholamines in the pathogenesis of TSS. Steroid hormones potentiate the actions of catecholamines and regulate and channelize catecholaminergic actions, thereby preventing their deleterious effects on the cardiac tissue [18, 19].

Beta blockage is the cornerstone of TSS treatment. Therefore, it is important to have a high suspicion level for pheochromocytoma, since beta blockage in PC may lead to unopposed alpha stimulation with important clinical implications [20]. Accordingly, treatment of these patients requires alpha and beta blockage, and, after hemodynamic stabilization, adrenalectomy.

## 4. Conclusion

Pheochromocytoma can present with complex, enigmatic, and rare clinical pictures, such as shock. Clinicians should be aware of this atypical presentation when managing adrenal masses.

## Figures and Tables

**Table 1:** Case 1 analytical results (at admission).

Hemoglobin	11.3 g/dL
Hematocrit	35.9%
Leucocytes	24.08∗10^9^/uL
Platelets	606∗10^9^/uL
Glucose	458 mg/dL
Creatinine	1.78 mg/dL
Urea	39 mg/dL
Sodium	133 mmol/L
Potassium	3.42 mmol/L
Chlorine	95.8 mmol/L
Total bilirubin	<0.15 mg/dL
Albumin	4 g/dL
Amylase	23 U/L
Lipase	31 U/L
TGP	13 U/L
Troponin	464 ng/L
NT pro-BNP	1264 pg/mL
Myoglobin	530 ng/mL
C-reactive protein	6.66 mg/dL
Beta hCG	<2 mIU/mL

**Table 2:** Case 2 analytical results (at admission).

Hemoglobin	10.4 g/dL
Hematocrit	32.9%
Leucocytes	22∗10^9^/uL
Platelets	390∗10^9^/uL
Creatinine	1 mg/dL
Urea	59 mg/dL
Sodium	145 mmol/L
Potassium	4.0 mmol/L
Chlorine	108 mmol/L
AST	476 U/L
ALT	482 U/L
Troponin	26147 ng/L
Myoglobin	283 ng/mL
C-reactive protein	83.4 mg/dL
Beta hCG	<1.2 mUI/mL

## Data Availability

The radiology images used during the current study are available from the corresponding author upon reasonable request.
